# Antibiotics Resistance and Adhesive Properties of Clinical *Staphylococcus aureus* Isolated from Wound Infections

**DOI:** 10.3390/microorganisms11051353

**Published:** 2023-05-22

**Authors:** Khulood Fahad Alabbosh, Tarek Zmantar, Abdulrahman S. Bazaid, Mejdi Snoussi, Emira Noumi

**Affiliations:** 1 Department of Biology, College of Science, University of Hail, Hail 2440, Saudi Arabia; m.snoussi@uoh.edu.sa; 2 Laboratory of Analysis, Treatment, Valorization of Environmental, and Product Pollutants, Faculty of Pharmacy of Monastir, University of Monastir, Monastir 5000, Tunisia; tarek.zmantar@fmdm.u-monastir.tn; 3 Department of Medical Laboratory Science, College of Applied Medical Sciences, University of Hail, Hail 55476, Saudi Arabia; ar.bazaid@uoh.edu.sa; 4 Laboratory of Genetics, Biodiversity and Valorization of Bio-Resources (LR11ES41), Higher Institute of Biotechnology of Monastir, University of Monastir, Avenue Tahar Haddad, BP74, Monastir 5000, Tunisia

**Keywords:** *Staphylococcus aureus*, antibiotic resistance, adhesion genes, biofilm, molecular approach

## Abstract

*Staphylococcus aureus* (*S. aureus*) is a ubiquitous pathogen responsible for several severe infections. This study aimed to investigate the adhesive properties and antibiotic resistance among clinical *S. aureus* isolated from Hail Hospital Province, Kingdom of Saudi Arabia (KSA), using molecular approaches. This study was conducted according to the ethical committee at Hail’s guidelines on twenty-four *S. aureus* isolates. A polymerase chain reaction (PCR) was performed to identify genes encoding the β-lactamase resistance (*bla*Z), methicillin resistance (*mec*A), fluoroquinolone resistance (*nor*A), nitric oxide reductase (*nor*B), fibronectin (*fnb*A and *fnb*B), clumping factor (*clf*A) and intracellular adhesion factors (*ica*A and *ica*D). This qualitative study tested adhesion based on exopolysaccharide production on Congo red agar (CRA) medium and biofilm formation on polystyrene by *S. aureus* strains. Among 24 isolates, the *cna* and blaz were the most prevalent (70.8%), followed by *nor*B (54.1%), *clf*A (50.0%), *nor*A (41.6%), *mec*A and *fnb*B (37.5%) and *fnb*A (33.3%). The presence of *ica*A/*ica*D genes was demonstrated in almost all tested strains in comparison to the reference strain, *S. aureus* ATCC 43300. The phenotypic study of adhesion showed that all tested strains had moderate biofilm-forming capacity on polystyrene and represented different morphotypes on a CRA medium. Five strains among the twenty-four harbored the four genes of resistance to antibiotics (*mec*A, *nor*A, *nor*B and *bla*z). Considering the genes of adhesion (*cna*, *clf*A, *fnb*A and *fnb*B), these genes were present in 25% of the tested isolates. Regarding the adhesive properties, the clinical isolates of *S. aureus* formed biofilm on polystyrene, and only one strain (S17) produced exopolysaccharides on Congo red agar. All these results contribute to an understanding that the pathogenesis of clinical *S. aureus* isolates is due to their antibiotic resistance and adhesion to medical material.

## 1. Introduction

Epidemiological surveys in clinical settings have revealed a significant increase in hospital infections [1,2]. This rise is linked to the use of immunosuppressive medications in treatment. *Staphylococcus* is the most common genus among the microorganisms involved [3]. Several *Staphylococcus* species are now responsible for severe infections with high morbidity and mortality [4]. The *Staphylococcus* genus contains 47 species and 24 subspecies, 17 of which are found in humans [2]. Its other species can be found in animals or food [5]. Three species are primarily responsible for human pathology among those isolated in humans: *Staphylococcus aureus*, *Staphylococcus epidermidis*, and *Staphylococcus saprophyticus* [6]. Of these, *S. aureus* is prevalent. These species are frequently found on human skin and in the nostrils. They are both commensal bacteria and important human pathogens. They are involved in various pathologies, including 1 to 5% of community infections and up to 30% of hospital infections [7].

*Staphylococcus aureus* is a bacterium that can infect both humans and animals. It can also result in food poisoning. Other sources of contamination include hospital equipment and surfaces and the food industry [4,8]. This germ has recently gained notoriety due to its resistance to antimicrobials, particularly methicillin (β-Lactamines). The presence of the exact clone of *S. aureus* in the anterior part of the nose and on the skin is frequently detected in colonized people, indicating an endogenous source of bacteria that can cause infections [4] or spread to other patients [9,10]. Hospital staff represent a second reservoir for *S. aureus* which can be transmitted to patients [11]. *S. aureus* strains’ high pathogenicity is related to their ability to resist multiple antimicrobials and adapt to changing environmental conditions [12]. This species’ virulence is associated with numerous virulence factors encoded by genes on the chromosome or plasmid 13, as well as with the combined action of various bacterial surface components [11,12]. This bacterial genus’ pathogenicity is due to its ability to adhere to host cells [13].

The formation of a biofilm contributes to the resistance to the immune defenses and antimicrobial agents [14]. Many genes are involved in adhesion and biofilm formation [15]. The ica ADBC gene, which produces biofilm formation by PIA, is found in all strains of *S. aureus*. Stress conditions such as anaerobic conditions, extreme temperature, ethanol, and antimicrobials regulate the ica gene. The ica ADBC locus is required for *Staphylococcus* biofilm formation [16]. Other proteins, such as FnbpA and Fnbp B, are required for biofilm formation. Indeed, there are two main FnBPs in *S. aureus*, FnBPA, and FnBPB. They are encoded by two closely related genes, *fnb*A, and *fnb*B, respectively [17].

Due to the increase in the rate of nosocomial infections with *S. aureus* strains and the prevalence of the multidrug-resistant strains, we aimed in this study to look into the biofilm potency and antibiotic resistance profile, and to explain theses virulence factors by the study of the distribution of the genes responsible for these mechanisms in clinical *S. aureus* strain genomes.

## 2. Materials and Methods

### 2.1. Tested Strains and Culture Conditions

This study was carried out by the Ethics Committee at Hail Affairs (reference: H-08-L-074). Patient privacy and data confidentiality were protected following the specifications of Helsinki Declaration. Patients developing wound infections were the subjects of this study, which was conducted in March 2021. For this, swab samples were collected from the depths of the wound using a sterile cotton swab under aseptic conditions. The isolates were transferred into a sterile nutrient broth in a test tube and were delivered to the microbiology laboratory at King Khalid Hospital in Hail, Saudi Arabia within 1 h. Twenty-four (24) *S. aureus* strains were studied in total. 

Blood and MacConkey agar plates (Bio-rad, Marnes-la-Coquette, France) were used to plate wound samples, which were then incubated at 37 °C for 24 h. Subculturing on mannitol salt agar was used to confirm the purity of the suspected *S. aureus* isolates (Bio-rad, Marnes-la-Coquette, France). Catalase, coagulase, and DNase enzymes were identified during the strain identification process. The reference strain of *S. aureus* ATCC 43300 was used as a quality control.

### 2.2. Study of Exopolysaccharide Production 

As previously described, the slime production of the ability pathogenic bacteria was evaluated by culturing each bacterium on Congo red agar (CRA) [18,19]. After aerobic incubation at 37 °C for 24 h, the results were interpreted as follows: very black and black colonies were considered to be regular slime-producing strains. Almost black, very red, red, and Bordeaux-colored colonies were classified as non-slime-producing strains [20,21].

### 2.3. Quantitative Biofilm Production Assay by S. aureus Cells 

A semi-quantitative technique performed using 96-well polystyrene plates (Nunc, Roskilde, Denmark) was used to produce biofilm by *S. aureus* strains in trypticase soya broth (TSB, Bio-rad, Marnes-la-Coquette, France) using the crystal violet 1% (CV) staining assay [22,23]. Bacterial cells were cultured for 24 h at 37 °C in TSB supplemented with 2% glucose (*w*/*v*). The optical density after the CV staining was measured at 570 nm (OD_570_) and the biofilm formation was interpreted as highly positive (OD_570_ ≥ 1), having low positivity (0.1 ≤ OD_570_ < 1), and negative (OD_570_ < 0.1) [24]. 

### 2.4. Detection of ica A and ica D loci, cna, fnbA, fnbB and clfA Adhesins Genes

The inoculated bacterial strains of *S. aureus* were incubated for 18 to 24 h at 37 °C in nutrient broth for bacterial DNA extraction. First, pure colonies were suspended in 1 mL of a Tris–EDTA (TE) solution. The cell suspension was washed by centrifugation at 13,200 rpm for 5 min and the pellet was suspended in 200 μL TE, vortexed, and then heated at 95 °C for 10 min. A final centrifugation was performed at 13,200 rpm for 5 min and the bacterial DNA supernatant was stored at −20 °C.

The detection of icaA and icaD genes in the tested strains’ genomes was also performed using the protocol described previously [25]. All the primers used, the PCR conditions, and the amplicon size are reported in Table 1. The strain of *S. aureus* ATCC 43300 was used as a positive control. The PCR conditions of *cna*, *fnb*A, and *fnb*B genes were accomplished as described elsewhere [24,26]. The *clf*A gene (1000 bp) responsible for the binding to fibrinogen was amplified by PCR according to the protocol previously described [27]. All PCR primers sequences are listed in Table 1.

### 2.5. PCR Amplification of Efflux Pump Genes 

Amplification conditions are summarized in Table 2. The annealing temperature was about 45 °C for nor A and 53 °C for nor B. The strain of *S. aureus* ATCC 43300 was used as a positive control.

### 2.6. Detection of mec A, blaZ, norA and norB Genes

Molecular detection of mec A and bla Z genes was performed using the forward and reverse primers presented in Table 2. PCR assessments were performed according to the specifications of Geha et al. [28] and Martineau et al. [29], respectively. The strain of *S. aureus* ATCC 43300 was used as a positive control.

### 2.7. Study of Antimicrobial Susceptibility Profile

The study of antibacterial resistance profiles was performed according to the protocol previously described by Bazaid et al. [30], using a BD Phoenix™ M50 instrument (Becton, Dickinson and Co., Franklin Lakes, NJ, USA). Twenty-two (22) antibiotics were tested: gentamicin, cefoxitin, cefotaxime, ceftaroline, ampicillin, penicillin G, oxacillin, daptomycin, trimethoprim, teicoplanin, vancomycin, clindamycin, erythromycin, linezolid, mupirocin, nitrofurantoin, ciprofloxacin, levofloxacin, moxifloxacin, rifampin, tetracycline, and tigecycline. The obtained data were analyzed and interpreted according to the guidelines of the Clinical Laboratory Standards Institute (CLSI).

## 3. Results

### 3.1. Qualitative and Quantitative Study of Biofilm Formation

**Figure 1:**
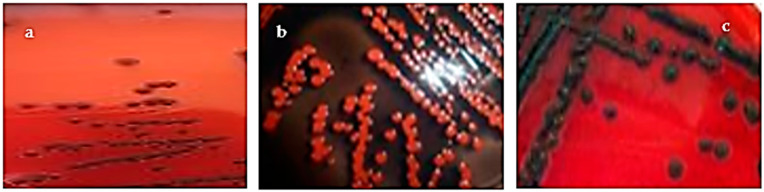
Different morphotypes obtained on Congo red agar based on the color obtained. (**a**) Bordeaux; (**b**) almost black; (**c**) very black (strain S17).

The capacity of clinical *S. aureus* strains to produce biofilm was estimated qualitatively by the culture on the CRA medium and quantitatively by the ability to create a biofilm on a polystyrene surface. In addition, three morph types were defined according to their color on CRA were obtained: non-slime-producing *S. aureus* strains characterized by Bordeaux (70.8%) and almost black colonies (25.0%). In contrast, a black colony characterizing slime-positive bacteria was obtained only for strain S17 of *S. aureus* (4.1%) (Figure 1, Table 3). 

**Figure 2:**
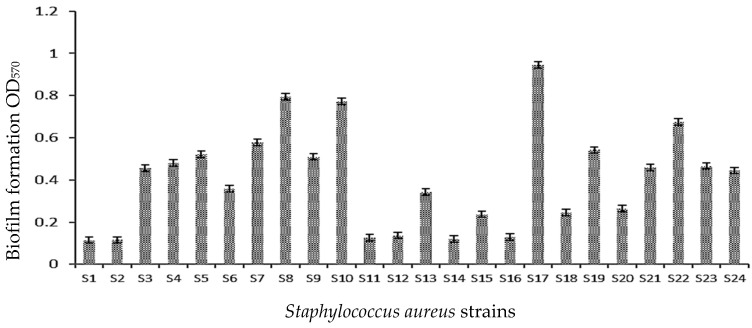
Biofilm formation (OD_570_) by *S. aureus* strains on 96-well polystyrene plates.

The main results showed that all *S. aureus* tested strains (also S17 very black on CRA) exhibited moderate biofilm formation on polystyrene (0.1 < OD_570_ < 1) compared to the reference strain *S. aureus* ATCC 43300 (OD_570_ = 1.89 ± 0.13) (Figure 2, Table 3).

According to our results, 19 *S. aureus* strains (79.1%) were positive for both *ica*A (188 bp) and *ica*D (198 bp) genes encoding the intracellular adhesins A and D compared to the positive control strain of *S. aureus* ATCC 43300 for both tested genes (Supplementary Material Figure S1, Table 3). In addition, all the primers used in the experiment exhibited specificity, with a single band. Therefore, only five clinical strains (20.8%) were expected from this correlation (Table 3).

### 3.2. Distribution of Adhesion Genes

This study emphasized four biofilm-related genes encoding fibronectin-binding proteins A and B (*fnb*A and *fnb*B), bound coagulase (*clf*A), and collagen adhesin gene (*cna*) involved in *S. aureus* cell attachment and multiplication. All *S. aureus* strains expressed biofilm genes. As shown in Table 4, the *fnb*A (259 bp) and *fnb*B (523 bp) encoding fibronectin were detected in 33.3% and 37.5%, respectively, of the total strains (Supplementary Material Figure S2). The *clf*A (288 bp) and *cna* (192 bp) genes encoding collagen were amplified in 50.0% and 70.8% of the total tested strains (Supplementary Material Figure S3).

As shown in Table 4, six *S. aureus* strains (S1, S8, S9, S13, S20, and S22) among 24 (25.0%) have the four tested genes (*cna*, *clf*A, *fnb*A, and *fnb*B) in their genomes compared to the reference strain *S. aureus* ATCC 43330.

### 3.3. Distribution of Antibiotic Resistance Genes

The β-lactamases *bla*Z gene (*bla*Z), methicillin resistance determinant (*mec*A), fluoroquinolones resistance gene (*nor*A), and nitric oxide reductase (*nor*B) gene were studied for all *S. aureus* isolates. All these results are presented in Supplementary Material Figure S4 and Table 5.

All the genomes studied in this work had blaz (70.8%), norB (54.1%), norA (41.6%), and mecA (37.5%) genes. The reference strain of *S. aureus* ATCC 43300 and the clinical isolates (S1, S8, S9, S23, and S24) had in common the four genes present in their genomes (Table 5).

Based on the results of the phenotypic profile of antibacterial resistance, the clinical strains of *S. aureus* S12, S22, and S2 were more resistant to the tested antibiotics, with percentages of resistance of 63.6%, 54.5%, and 45.4%, respectively. Compared to the more sensitive strains, S10 was very susceptible to the action of the antibiotics (0.9% of resistance), followed by isolate S17 (9.1% of resistance) (Supplementary Material Table S1).

## 4. Discussion

The skin surface can be affected by several microorganisms that cause wound infection. *S. aureus* is a pathogenic bacterium that is resistant to penicillin due to the production of β-lactamase and which contributes to the inhibition of its antibacterial activity. *S. aureus* causes many problems in hospitals and is usually resistant to antimicrobials [3,32].

In the present study, antibiotic susceptibility results were reported to be influenced by various factors, such as the expression of the antimicrobials resistance genes. Our results showed that the *bla*Z gene was widely spread among *S. aureus* strains (70.83%), followed by *nor*A, *nor*B, and *mec*A genes. The *nor*B gene has a single antimicrobial resistance mechanism. At the same time, *mec*A and *bla*Z possess several antibiotic resistance mechanisms [3,17,32].

Efflux pump complexes and resistance-conferring antibiotic subunits, as well as protein(s) (*no*rA and *nor*B), are the most prevalent mechanisms of antibiotic resistance. Other instruments include the antibiotic resistance gene or operon (*mec*A and *bla*Z) and antibiotic inactivation enzyme (SAT4 and mphC) [31].

*S. aureus* strains express resistance genes from external sources [33,34]. This can be natural or due to antimicrobial abuse and misuse, leading to chromosomal mutation and antibiotic selection. Antibiotic-resistant strains are receiving significant consideration in the contemporary era [26]. Resistance to many antimicrobial agents causes critical problems in treating *S. aureus* infection [35,36]. For example, methicillin resistance contributes to the inhibition of the synthesis of the cell wall. 

*S. aureus* strains can live in biofilms in their natural environment, where planktonic cells proliferate and accumulate in multilayer cells. This structure can protect microorganisms from the action of antimicrobials, extracellular enzymes, and stress factors [24].

The results of the biofilm formation using the crystal violet method showed that all tested *S. aureus* strains formed a biofilm on the polystyrene. Mathur et al. [37] demonstrated that 14.47% of isolates from blood, medical devices, and skin surfaces formed a strong biofilm, 39.4% formed a moderate biofilm, and 46% of the strains were not biofilm producers [37].

The ability of clinical *S. aureus* isolates to produce exopolysaccharides on CRA plates showed that only one strain was able to produce mucus compared to the results obtained by Arciola et al., in which 57.5% of the isolates had mucus [20].

Bacterial biofilm formation is encoded by adhesions responsible for the pathogenicity of *S. aureus* strains [20]. Therefore, in this study, we focused on detecting genes coding for them.

The *ica* A and *ica* D genes responsible for the synthesis of polysaccharide intercellular adhesin (PIA) were detected in all strains. The presence of *ica*A/*ica*D genes was detected in 19/24 *S. aureus* strains (79.16%). Arciola et al. [26] found that 60.86% of *S. aureus* strains harbor *ica*A and *ica*D genes. In previous research, it has been demonstrated that 98% of clinical isolates of *S. aureus* have *ica*A, and 96% have *ica*D [38].

In the study of Rohde et al. [25], all tested *S. aureus* strains, including slime-negative isolates, expressed the *ica*A gene. This was contrary to the research of Arciola et al. [26], who correlate the presence of *ica*A/*ica*D genes to the production of exopolysaccharides. 

Adhesin genes (*cna*, *fnb*A, *fnb*B and *clf*A) were expressed in six *S. aureus* strains (25%). This demonstrated that *clf*A and *clf*B proteins were implicated in the pathogenesis of *S. aureus*, conferring endocarditis, bacteremia, or pyonephrosis [20]. Our results are similar to those reported by Arciola et al. [26], who noted that 84/191 clinical *S. aureus* strains (44%) expressed two adhesins genes (*fnb*A and *cna*). The *cna* gene was detected in 70.83% of the tested strains in the present work. 

Several virulence factors of *S. aureus*, including fibronectin-binding (*fnb*A and *fnb*B), responsible of bacterial adhesion have been largely described [38,39].

## 5. Conclusions

This work provides new information on *S. aureus* isolated from wound surfaces. In fact, *S. aureus* strains are biofilm producers. They show high percentages of genes responsible for its adhesion and resistance to antimicrobials. These genes can make strains capable of colonizing many human organs. In further work, we envisage studying the effect of natural and synthetic compounds on the biofilm and antimicrobial resistance of *S. aureus* strains using molecular and in silico approaches.

## Figures and Tables

**Table 1:** Primers used for detection of adhesion genes.

Gene	Primer Sequence 5′-3′	Amplicon Size (bp)	References
*ica*A	ACACTTGCTGGCGCAGTCAA TCTGGAACCAACATCCAACA	188	[27]
*ica*D	ATGGTCAAGCCCAGACAGAG AGTATTTTCAATGTTTAAAGCAA	198	[27]
*cna*	AAAGCGTTGCCTAGTGGAGA AGTGCCTTCCCAAACCTTTT	192	[28]
*fnb*A	GATACAAACCCAGGTGGTGG TGTGCTTGACCATGCTCTTC	191	[28]
*fnb*B	TGTGCTTGACCATGCTCTTC AGTTGATGTCGCGCTGTATG	201	[28]
*clf*A	CCGGATCCGTAGCTGCAGATGCACC GCTCTAGATCACTCATCAGGTTGTTCAGG	1000	[29]

**Table 2:** Primers used for the detection of genes encoding antimicrobials resistance.

Gene	Primer Sequence 5′-3′	Amplicon Size (bp)	References
*mec*A	AACAGGTGAATTATTAGCACTTGTAAG ATTGCTGTTAATATTTTTTGAGTTGA	140	[30]
*nor*A	TTCACCAAGCCATCAAAAAG CTTGCCTTTCTCCAGCAATA	620	[31]
*nor*B	AGCGCGTTGTCTATCTTTCC GCAGGTGGTCTTGCTGATAA	213	[31]
*bla*Z	ACTTCAACACCTGCTGCTTTC TGACCACTTTTATCAGCAACC	172	[32]

**Table 3:** Correlation between phenotypic and genotypic adhesive properties of clinical *S. aureus* strains.

Strains	OD_570_ ± SD	Biofilm Production	Phenotype on CRA	Slime	*ica A*/*icaD* Gene
*S. aureus* ATCC 43300	1.89 ± 0.13	High biofilm	Very Black	S+	*ica*A+/*ica*D +
S1	0.11 ± 0.01	Moderate biofilm	Bordeaux	S−	*ica*A+/*ica*D +
S2	0.11 ± 0.01	Moderate biofilm	Almost black	S−	*ica*A+/*ica*D +
S3	0.45 ± 0.01	Moderate biofilm	Bordeaux	S−	*ica*A+/*ica*D +
S4	0.48 ± 0.04	Moderate biofilm	Almost black	S−	*ica*A+/*ica*D +
S5	0.52 ± 0.01	Moderate biofilm	Bordeaux	S−	*ica*A+/*ica*D +
S6	0.35 ± 0.03	Moderate biofilm	Almost black	S−	*ica*A+/*ica*D +
S7	0.57 ± 0.04	Moderate biofilm	Bordeaux	S−	*ica*A+/*ica*D +
S8	0.79 ± 0.01	Moderate biofilm	Almost black	S−	*ica*A+/*ica*D +
S9	0.51 ± 0.01	Moderate biofilm	Bordeaux	S−	*ica*A+/*ica*D +
S10	0.77 ± 0.02	Moderate biofilm	Almost black	S−	*ica*A+/*ica*D +
S11	0.12 ± 0.01	Moderate biofilm	Bordeaux	S−	*ica*A −/*ica*D −
S12	0.13 ± 0.01	Moderate biofilm	Bordeaux	S−	*ica*A −/*ica*D −
S13	0.34 ± 0.01	Moderate biofilm	Bordeaux	S−	*ica*A+/*ica*D +
S14	0.12 ± 0.01	Moderate biofilm	Bordeaux	S−	*ica*A −/*ica*D −
S15	0.23 ± 0.01	Moderate biofilm	Bordeaux	S−	*ica*A+/*ica*D +
S16	0.13 ± 0.03	Moderate biofilm	Almost black	S−	*ica*A −/*ica*D −
S17	0.94 ± 0.04	Moderate biofilm	Very Black	S+	*ica*A+/*ica*D +
S18	0.24 ± 0.02	Moderate biofilm	Bordeaux	S−	*ica*A −/*ica*D −
S19	0.54 ± 0.01	Moderate biofilm	Bordeaux	S−	*ica*A+/*ica*D +
S20	0.26 ± 0.03	Moderate biofilm	Bordeaux	S−	*ica*A+/*ica*D +
S21	0.45 ± 0.01	Moderate biofilm	Bordeaux	S−	*ica*A+/*ica*D +
S22	0.67 ± 0.02	Moderate biofilm	Bordeaux	S−	*ica*A+/*ica*D +
S23	0.46 ± 0.02	Moderate biofilm	Bordeaux	S−	*ica*A+/*ica*D +
S24	0.44 ± 0.02	Moderate biofilm	Bordeaux	S−	*ica*A+/*ica*D +
% of positivity				8.33%	83.33%

S+: slime producer; S−: slime non producer.

**Table 4:** Distribution of adhesion genes *cna, clfA*, *fnbA* and *fnbB* in *S. aureus* strains genome.

Strains	*cna*	*clfA*	*fnbA*	*fnbB*	% of the Presence of the Four Genes
*S. aureus* ATCC 43300	+	+	+	+	100%
S1	+	+	+	+	100%
S2	−	−	−	−	0%
S3	+	+	−	+	75%
S4	+	−	−	+	50%
S5	+	−	−	+	50%
S6	+	−	−	−	25%
S7	−	−	−	−	0%
S8	+	+	+	+	100%
S9	+	+	+	+	100%
S10	−	−	+	−	25%
S11	−	−	−	−	0%
S12	−	−	−	−	0%
S13	+	+	+	+	100%
S14	−	−	−	−	0%
S15	+	−	−	−	25%
S16	−	−	−	−	0%
S17	+	+	−	−	50%
S18	+	+	−	−	50%
S19	+	−	−	−	25%
S20	+	+	+	+	100%
S21	+	+	−	−	50%
S22	+	+	+	+	100%
S23	+	+	−	−	50%
S24	+	+	+	−	75%
% of positivity	75%	54.16%	37.5%	41.66%	

**Table 5:** Distribution of antibiotic resistance genes mec A, norA, norB and blaz in *S. aureus* strains genome.

Strains	*mecA*	*norA*	*norB*	*blaZ*	% of the Presence of the Four Genes
*S. aureus* ATCC	+	+	+	+	100%
S1	+	+	+	+	100%
S2	−	−	−	−	0%
S3	+	−	−	+	50%
S4	+	−	+	−	50%
S5	+	−	−	+	50%
S6	+	−	+	+	75%
S7	−	−	−	+	25%
S8	+	+	+	+	100%
S9	+	+	+	+	100%
S10	−	−	−	−	0%
S11	−	−	+	+	50%
S12	−	−	−	+	25%
S13	−	+	+	−	50%
S14	−	−	−	+	25%
S15	−	+	+	−	50%
S16	−	−	−	+	25%
S17	−	−	−	−	0%
S18	−	−	−	−	0%
S19	−	−	−	+	25%
S20	−	+	+	+	75%
S21	−	+	+	+	75%
S22	−	+	+	+	75%
S23	+	+	+	+	100%
S24	+	+	+	+	100%
% of positivity	41.66%	45.83%	58.33%	75%	

## Data Availability

Not applicable.

## Supplementary Materials

The following supporting information can be downloaded at: https://www.mdpi.com/article/10.3390/microorganisms11051353/s1, Figure S1: Agarose gel electrophoresis of PCR amplification of *ica*A (188 bp) and *ica*D (198 bp) locus. Lane 1: 100 pb DNA molecular size marker; Lanes 3-5: PCR amplification of *ica*A; Lanes 6-8: PCR amplification of *ica*D. Lane 2: Negative control; Lanes 3 to 8: PCR amplicons obtained with DNA of *S. aureus*. Lane 3: *S. aureus* ATCC 43300, Lane 4: S1; Lane 5: S3; Lane 6: S4; Lane 7: S8; Lane 8: S10. Figure S2: Agarose gel electrophoresis of PCR amplification of *fnb*A gene (191 bp) and *fnb*B gene (201 bp). Lane 1: 100 bp DNA molecular size marker; Lane 2: negative control; Lanes 3-5: PCR amplification of *fnb*A gene; Lanes 6-7: PCR amplification of *fnb*B gene; Lane 3 to 7: PCR amplicons obtained with DNA amplification of *S. aureus*; Lane 3: *S. aureus* ATCC 43300; Lane 4: S8; Lane 5: S13; Lane 6: *S. aureus* ATCC 43300; Lane 7: S20. Figure S3: Agarose gel electrophoresis of polymerase chain reaction (PCR) amplification *cna* gene (192 bp) and *clf*A gene (1kb). Lane 1, 100 bp DNA molecular size marker. Lane 2 negative control; Lanes 3-6 PCR amplification of *cna* gene, lanes 7-8 PCR amplification of *clf*A gene. Lane 3 to 8 PCR amplicons obtained with DNA amplification of *S. aureus*. Lane 3, *S. aureus* ATCC 43300; Lane 4, S1; Lane 5, S6; Lane 6, S15; Lane 7, *S. aureus* ATCC 43300; Lane 8, S9. Figure S4: Agarose gel electrophoresis of polymerase chain reaction (PCR) amplification of *mec*A (140 pb), *nor*A (620 bp), *nor*B (213 bp) and *bla*z (172 bp) genes. Lane 1: 100 bp DNA molecular size marker; Lanes 2-3 PCR amplification of *mec*A gene. Lanes 4-5 PCR amplification of *nor*A gene. Lanes 6-7 PCR amplification of *nor*B gene and lane 8 PCR amplification of *bla*z gene. Lanes 2: *S. aureus* ATCC 43300; Lanes 3 to 8 PCR amplicons obtained with DNA amplification of *S. aureus*. Lane 2: S1; Lane 3: S3; Lane 4: S8; Lane 5: S9; Lane 6: S6; Lane 7: S11; Lane 8: S23. Table S1: Study of antimicrobial susceptibility profile of clinical *S. aureus* strains.
