# Supplementary material for: Antibiotics Resistance and Adhesive Properties of Clinical Staphylococcus aureus Isolated from Wound Infections
Source: Microorganisms. 2023 May 22;11(5):1353. doi: 10.3390/microorganisms11051353 (PMC10221594; doi:10.3390/microorganisms11051353)
Supplement: Supplementary file 1 [file microorganisms-11-01353-s001.zip › microorganisms-2393988-supplementary.pdf]

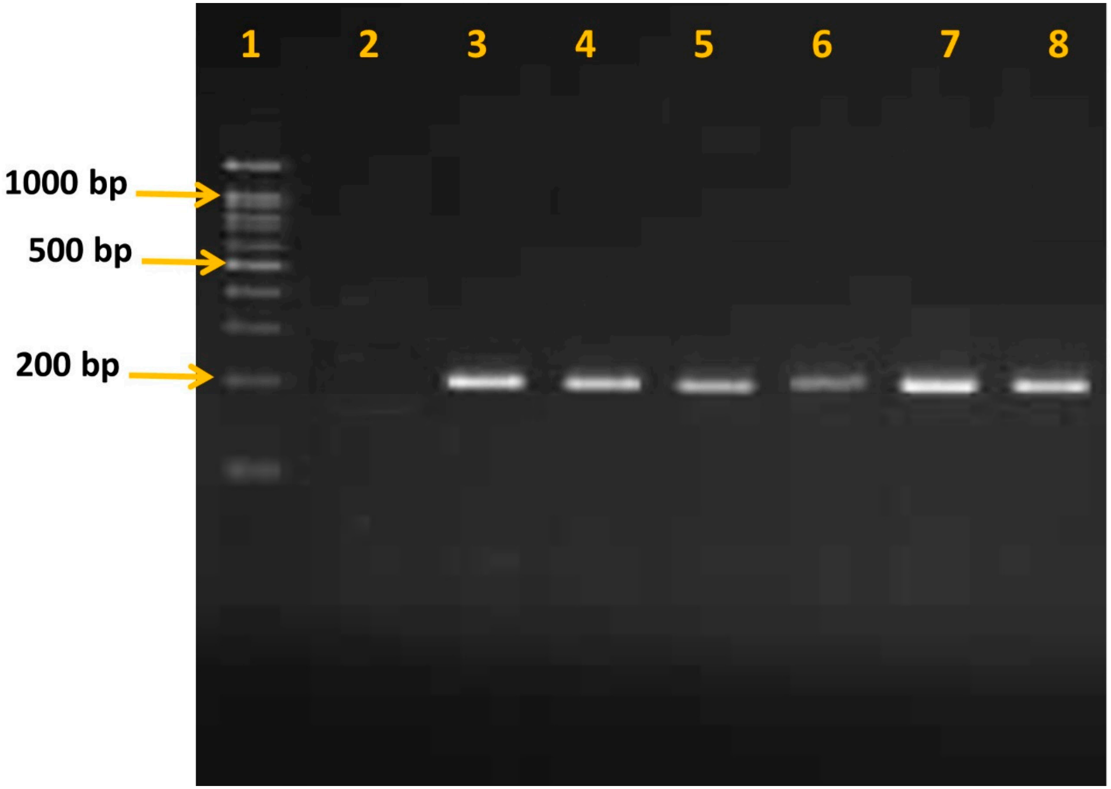

Supplementary material Figure S1. Agarose gel electrophoresis of PCR amplification of *ica*A (188 bp) and *ica*D (198 bp) locus. Lane 1: 100 pb DNA molecular size marker; Lanes 3-5: PCR amplification of *ica*A; Lanes 6-8: PCR amplification of *ica*D. Lane 2: Negative control; Lanes 3 to 8: PCR amplicons obtained with DNA of *S. aureus*. Lane 3: *S. aureus* ATCC 43300, Lane 4: S1; Lane 5: S3; Lane 6: S4; Lane 7: S8; Lane 8: S10.

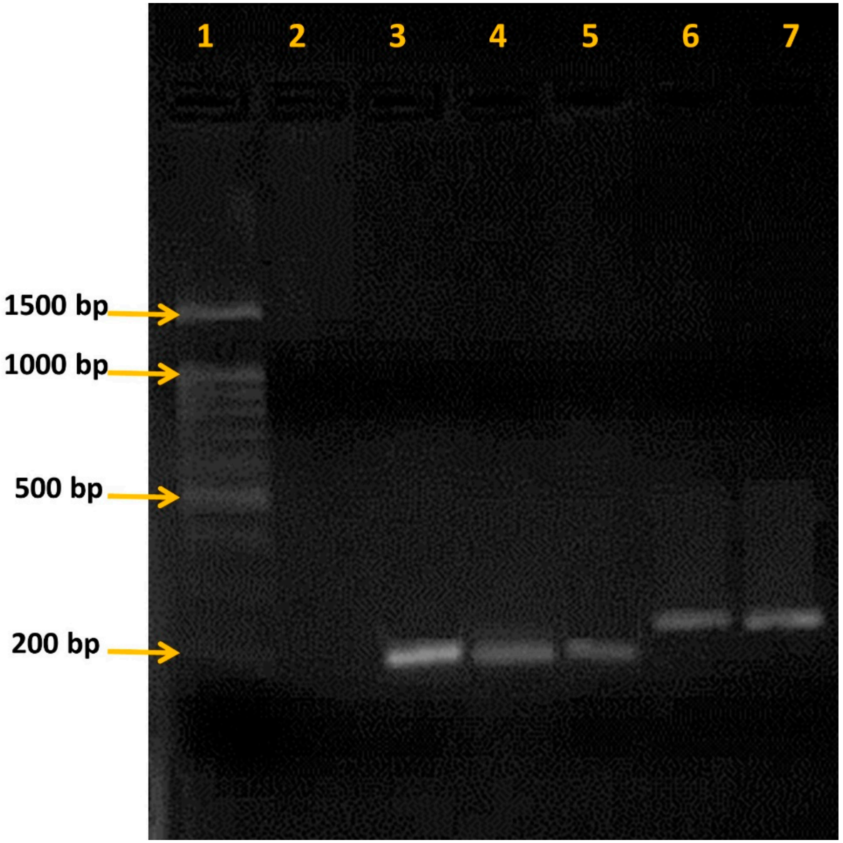

Supplementary material Figure S2. Agarose gel electrophoresis of PCR amplification of *fnb*A gene (191 bp) and *fnb*B gene (201 bp). Lane 1: 100 bp DNA molecular size marker; Lane 2: negative control; Lanes 3-5: PCR amplification of *fnb*A gene; Lanes 6-7: PCR amplification of *fnb*B gene; Lane 3 to 7: PCR amplicons obtained with DNA amplification of *S. aureus*; Lane 3: *S. aureus* ATCC 43300; Lane 4: S8; Lane 5: S13; Lane 6: *S. aureus* ATCC 43300; Lane 7: S20.

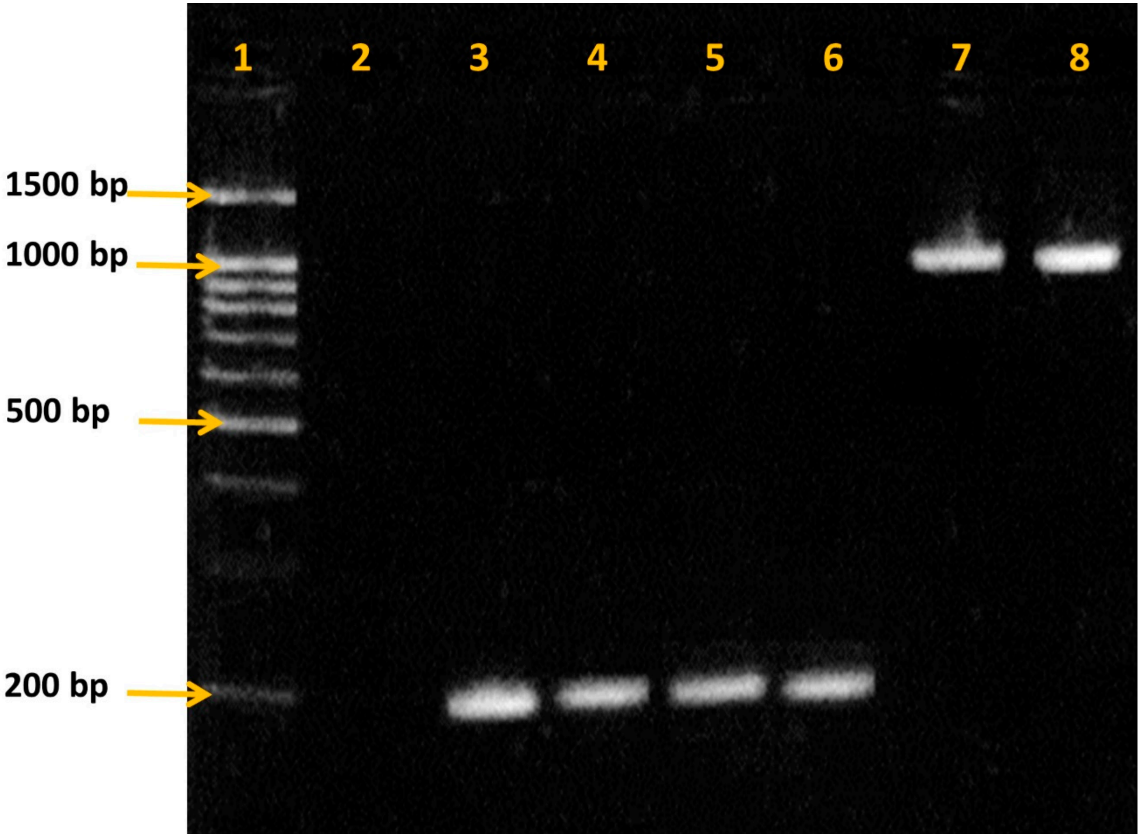

Supplementary material Figure S3. Agarose gel electrophoresis of polymerase chain reaction (PCR) amplification *cna* gene (192 bp) and *clf*A gene (1kb). Lane 1, 100 bp DNA molecular size marker. Lane 2 negative control; Lanes 3-6 PCR amplification of *cna* gene, lanes 7-8 PCR amplification of *clf*A gene. Lane 3 to 8 PCR amplicons obtained with DNA amplification of *S. aureus*. Lane 3, *S. aureus* ATCC 43300; Lane 4, S1; Lane 5, S6; Lane 6, S15; Lane 7, *S. aureus* ATCC 43300; Lane 8, S9.

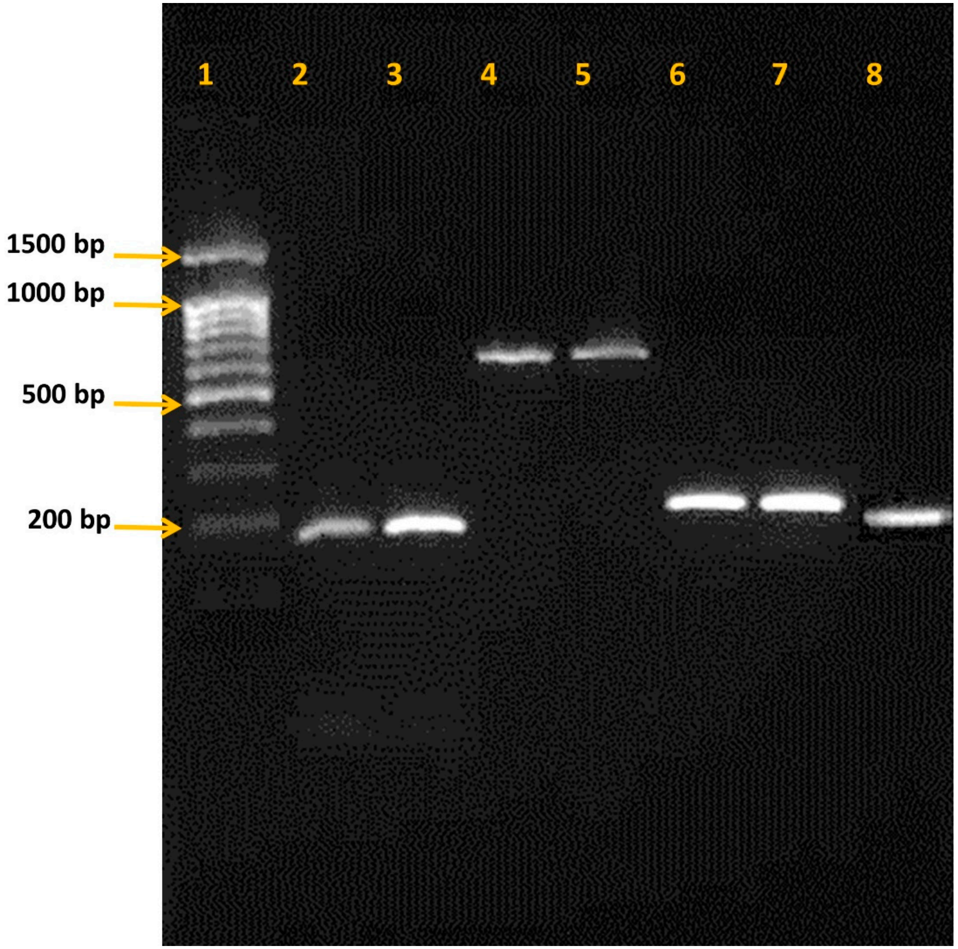

Supplementary material Figure S4. Agarose gel electrophoresis of polymerase chain reaction (PCR) amplification of *mec*A (140 pb), *nor*A (620 bp), *Nor*B (213 bp) and *blaz* (172 bp) genes. Lane 1: 100 bp DNA molecular size marker; Lanes 2-3 PCR amplification of *mec*A gene. Lanes 4-5 PCR amplification of *nor*A gene. Lanes 6-7 PCR amplification of *nor*B gene and lane 8 PCR amplification of *blaz* gene. Lanes 2: *S. aureus* ATCC 43300; Lanes 3 to 8 PCR amplicons obtained with DNA amplification of *S. aureus*. Lane 2: S1; Lane 3: S3; Lane 4: S8; Lane 5: S9; Lane 6: S6; Lane 7: S11; Lane 8: S23.

Supplementary material Table S1. Study of antimicrobial susceptibility profile of clinical *S. aureus* strains

**Antibiotics**	***Staphylococcus aureus* strains**																							
	**S1**		**S2**		**S3**		**S4**		**S5**		**S6**		**S7**		**S8**		**S9**		**S10**		**S11**		**S12**	
	**MIC**	**I**	**MIC**	**I**	**MIC**	**I**	**MIC**	**I**	**MIC**	**I**	**MIC**	**I**	**MIC**	**I**	**MIC**	**I**	**MIC**	**I**	**MIC**	**I**	**MIC**	**I**	**MIC**	**I**
**Gentamicin**	<2	S	>8	R	<2	S	2	S	2	S	2	S	2	S	>8	R	2	R	2	S	>0.5	R	2	S
**Cefoxitin**	<2	R	>8	R	>8	R	>8	R	>8	R	>8	R	>8	R	8	R	8	R	2	S	>8	R	>8	R
**Cefotaxime**	<8	R	8	R	8	R	>32	R	>32	R	>32	R	8	R	8	R	8	R	2	S	8	R	>32	R
**Ceftaroline**	<0.25	S	0.5	S	0.5	S	1	S	1	S	1	S	0.5	S	0.5	S	0.5	S	8	S	8	S	>2	R
**Ampicillin**	1	R	>1	R	1	R	>1	R	>1	R	>1	R	1	R	1	R	1	R	1	R	1	R	1	R
**Penicillin G**	1	R	>1	R	0.5	R	>1	R	>1	R	>1	R	>1	R	0.5	R	1	R	0.25	S	0.25	S	>1	R
**Oxacillin**	>2	R	>2	R	>2	R	>2	R	>2	R	>2	R	>2	R	2	S	2	R	0.25	S	0.5	R	>2	R
**Daptomycin**	1	S	1	S	1	S	1	S	1	S	1	S	1	S	1	S	1	S	1	S	1	S	1	S
**Trimethoprim**	>4	R	1	S	1	S	1	S	1	S	1	S	1	S	1	S	1	S	1	S	<10	S	1	S
**Teicoplanin**	8	S	>16	R	1	S	1	S	1	S	1	S	1	S	1	S	1	S	1	S	<0.25	S	2	S
**Vancomycin**	<1	S	1	S	1	S	1	S	1	S	1	S	1	S	1	S	1	S	1	S	1	S	1	S
**Clindamycin**	<0.5	S	>2	R	0.5	S	0.5	S	0.5	S	0.5	S	0.5	S	0.5	S	0.5	S	0.5	S	<0.25	S	>2	R
**Erythromycin**	>4	R	>4	R	0.25	S	>4	R	>4	R	0.25	S	>4	R	0.25	S	0.25	S	>4	R	>8	R	>4	R
**Linezolid**	4	S	1	S	1	S	1	S	1	S	1	S	1	S	1	S	1	S	1	S	2	S	1	S
**Mupirocin High level**	<256	S	256	S	256	S	256	S	256	S	256	S	256	S	256	S	256	S	<256	S	<256	S	256	S
**Nitrofurantoin**	<16	S	16	S	16	S	16	S	16	S	16	S	16	S	16	S	16	S	16	S	<16	S	32	S
**Ciprofloxacin**	2	R	0.5	S	0.5	S	0.5	S	0.5	S	0.5	S	0.5	S	0.5	S	0.5	S	0.5	S	0.5	S	>2	R
**Levofloxacin**	<1	S	1	S	1	S	1	S	1	S	1	S	1	S	1	S	1	S	0.5	S	0.25	S	>4	R
**Moxifloxacin**	<0.5	S	0.5	S	0.5	S	0.5	S	0.5	S	0.5	S	0.5	S	0.5	S	0.5	S	0.5	S	<0.25	S	>2	R
**Rifampin**	<0.5	S	0.5	S	0.5	S	0.5	S	0.5	S	0.5	S	0.5	S	0.5	S	0.5	S	0.5	S	<0.5	S	>2	R
**Tetracycline**	>8	R	>8	R	0.5	S	0.5	S	0.5	S	0.5	S	>8	R	0.5	S	0.5	S	0.5	S	<1	S	8	R
**Tigecycline**	<0.25	S	0.25	S	0.25	S	0.25	S	0.25	S	0.5	S	0.25	S	0.5	S	0.25	S	0.5	S	<0.12	S	0.25	R
% of Resistance		**40.9**		**45.4**		**22.7**		**27.2**		**27.2**		**22.7**		**31.8**		**22.7**		**27.2**		**0.9**		**27.2**		**63.6**
**Antibiotics**	**S13**		**S14**		**S15**		**S16**		**S17**		**S18**		**S19**		**S20**		**S21**		**S22**		**S23**		**S24**	
	**MIC**	**I**	**MIC**	**I**	**MIC**	**I**	**MIC**	**I**	**MIC**	**I**	**MIC**	**I**	**MIC**	**I**	**MIC**	**I**	**MIC**	**I**	**MIC**	**I**	**MIC**	**I**	**MIC**	**I**
**Gentamicin**	>0.5	R	<2	S	<2	S	<2	S	<2	S	>16	R	<0.5	S	<2	S	<2	S	<2	S	<2	S	>8	R
**Cefoxitin**	>8	R	<4	S	<2	S	<2	R	<2	S	<2	S	<2	S	>8	R	<2	R	>8	R	>8	R	8	R
**Cefotaxime**	8	R	8	R	<2	S	8	R	<2	S	8	R	8	R	<8	R	>8	R	<8	R	32	R	<8	R
**Ceftaroline**	<8	R	<8	R	16	R	<8	R	<8	R	<8	R	<8	R	>2	R	>32	R	2	I	1	S	>2	R
**Ampicillin**	>16	R	>16	R	>16	R	>16	R	>16	R	>16	R	>16	R	>1	R	>1	R	>1	R	>1	R	>1	R
**Penicillin G**	>25	R	<1	S	>25	R	>25	R	0.13	S	>25	R	>25	R	>1	R	>0.25	R	>1	R	1	R	>1	R

**Oxacillin**	0.5	R	<1	S	0.5	S	>2	R	<0.25	S	>4	R	>4	R	0.5	S	>2	R	0.5	S	>2	R	>2	R
**Daptomycin**	1	S	<1	S	1	S	2	R	<1	S	<1	S	<1	S	<1	S	>4	R	<1	S	<1	S	1	S
**Trimethoprim**	<10	S	<1	S	1	S	<1	S	<1	S	40	R	<10	S	<1	S	<1	R	<1	S	<1	S	1	S
**Teicoplanin**	<0.25	S	<1	S	>4	R	>4	R	<1	S	8	R	>32	R	>16	R	1	S	>16	R	<1	S	1	S
**Vancomycin**	<0.25	S	<1	S	<1	S	<1	S	1	S	1	S	>32	R	>16	R	<0.5	R	>16	R	<1	S	0.5	S
**Clindamycin**	<0.25	S	<0.25	S	1	S	1	S	<0.5	S	>8	R	>8	R	>2	R	<0.25	S	>2	R	<0.5	R	0.25	S
**Erythromycin**	>8	R	>8	R	<0.5	S	<0.5	S	<0.25	S	>8	R	>8	R	>4	R	<1	S	>4	R	>4	R	1	S
**Linezolid**	2	S	2	S	0.5	S	0.5	S	<1	S	1	S	1	S	>4	R	2	S	2	S	<1	S	2	S
**Mupirocin High level**	<1	S	<1	S	<1	S	8	R	2	S	<1	S	<1	S	<256	S	<256	S	<256	S	<256	S	256	S
**Nitrofurantoin**	<16	S	<16	S	2	S	2	S	2	S	<16	S	<16	S	<16	S	<16	S	<16	S	<16	S	16	S
**Ciprofloxacin**	<0.5	S	<0.5	S	256	S	256	S	256	S	<0.5	S	<0.5	S	<0.5	S	<0.5	S	>2	R	<0.5	S	0.5	S
**Levofloxacin**	0.25	S	<1	S	16	S	16	S	16	S	>8	R	<0.12	S	<1	S	<1	S	>4	R	<1	S	1	S
**Moxifloxacin**	<0.25	S	<0.25	S	0.5	S	>2	R	0.5	S	2	R	<0.25	S	<0.5	S	<0.5	S	2	R	<0.5	S	0.5	S
**Rifampin**	<0.5	S	<0.5	S	0.5	S	2	S	0.5	S	<0.5	S	>32	R	<0.5	S	<0.5	S	1	S	<0.5	S	0.5	S
**Tetracycline**	<1	S	<1	S	0.5	S	0.5	S	0.5	S	<1	S	<1	S	0.5	R	<0.5	S	>8	R	<0.5	S	>8	R
**Tigecycline**	<0.12	S	<1	S	0.5	S	0.5	S	0.5	S	<0.12	S	<0.12	S	0.5	S	<0.5	S	<0.25	S	<0.25	S	<0.25	S
**% of Resistance**		**36.3**		**18.1**		**18.1**		**45.4**		**9.1**		**54.5**		**45.4**		**50**		**40.9**		**54.5**		**31.8**		**36.3**
